# Comparative Analysis of Orthodontic Treatment Outcomes With Different Extraction Patterns in Identical Twins: A Case Report With Long-Term Follow-Up

**DOI:** 10.1155/crid/7856721

**Published:** 2025-10-06

**Authors:** Kun Li, Tiejun Wang, Qian Wang, Ning Li

**Affiliations:** ^1^Fushan Division, The Affiliated Yantai Stomatological Hospital, Binzhou Medical University, Yantai, China; ^2^Department of Orthodontics, The Affiliated Yantai Stomatological Hospital, Binzhou Medical University, Yantai, China

**Keywords:** comparative analysis, four-premolar extraction, identical twins treated differently, orthodontics, two-premolar extraction

## Abstract

Orthodontic treatment frequently involves tooth extractions to resolve dental crowding or protrusion and to enhance facial esthetics. The decision on which teeth to extract depends on various factors, including the degree of crowding, the patient's facial growth pattern, and the treatment objectives. The conventional (four-premolar) extraction pattern typically entails the removal of one premolar from each quadrant. However, in specific cases, the single-arch (two-premolar) extraction strategy, involving the symmetrical extraction of two maxillary premolars, can effectively resolve malocclusions with less invasiveness. A pair of identical twins sought orthodontic treatment to address dental crowding. Both patients exhibited nearly identical malocclusions, with severe crowding in the upper arch and mild or no crowding in the lower arch. Each twin underwent orthodontic treatment with a distinct extraction pattern: one received a four-premolar extraction method, while the other was treated with a two-premolar extraction pattern. The treatment outcomes demonstrated that both extraction strategies yielded satisfactory therapeutic effects, including well-aligned dentitions, stable occlusion, and balanced facial profiles. However, there were notable differences in the changes to the torque of anterior teeth between the twins. This case report concludes that while both four- and two-premolar extraction methods can achieve desirable treatment outcomes, their indications differ. The two-premolar extraction approach has a narrowed range of applicability but can be effective when used in appropriately selected cases.

## 1. Introduction

Class II malocclusion is characterized by a sagittal discrepancy between the upper and lower dental arches or jaws, with the lower arch or mandible positioned relatively distally, resulting in a Class II molar relationship. Orthodontic protocols for correcting this type of malocclusion are tailored to the patient's specific condition and can be divided into nonextraction and extraction approaches [1–4]. The extraction approach can further be categorized into conventional (four-premolar) extraction and single-arch (two-premolar) extraction strategies.

In recent years, the orthodontic community has been engaged in a vibrant debate regarding the relative merits of four- and two-premolar extraction strategies [5–8]. The four-premolar extraction approach is typically indicated for significant crowding in both the upper and lower arches, whereas the two-premolar extraction approach is primarily recommended when there are no cephalometric abnormalities or crowding in the lower arch [9–11]. The two-premolar extraction method is frequently employed in contemporary clinical practice due to its potential to reduce overall treatment duration and invasiveness while achieving effective therapeutic outcomes for the correction of complete Class II malocclusion [12–14]. Additionally, studies have shown that the two-premolar extraction offers similar long-term posttreatment stability and a higher success rate compared to the four-premolar extraction.

Identical twins share a strong genetic inheritance, often resulting in similar skeletal structures and soft tissue characteristics [15]. Twin studies are commonly employed to investigate the precise etiology of malocclusions, considering both genetic and environmental influences [16]. Despite their genetic similarities, the clinical orthodontic treatment protocols for a pair of identical twins can vary due to subtle differences in the anatomy of their stomatognathic system. Current literature lacks comparative studies of different extraction strategies in the treatment of identical twins with similar Class II malocclusion. This case report is aimed at filling that void by presenting a comparative analysis of orthodontic treatment outcomes in a set of identical twins, each undergoing different extraction patterns. One twin received the conventional four-premolar extraction, while the other was treated with a two-premolar extraction strategy. This comparison seeks to elucidate the differences in treatment outcomes, including tooth alignment, occlusion stability, and changes in facial profile, thereby contributing to the ongoing discussion regarding the efficacy of various extraction strategies in orthodontics.

## 2. Case Report

The patients, a pair of 12-year-old identical twins, presented to the orthodontic department seeking treatment for dental crowding. The patients had no history of systemic diseases.

**Figure 1:**
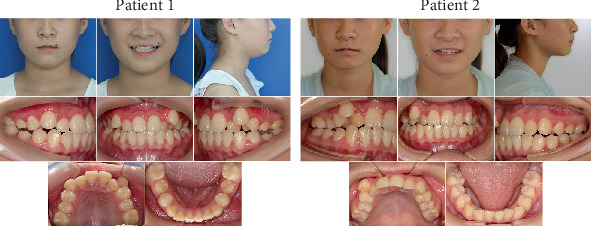
Facial and intraoral photographs taken at the initial visit.

Due to the patients being identical twins, they exhibited nearly identical facial and intraoral characteristics (Figure 1). They presented nearly balanced facial profiles, with both showing severe maxillary crowding. In the mandible, Patient 1 exhibited slight spacing, whereas Patient 2 showed mild crowding. Additionally, both patients exhibited bilateral Class II canine and molar relationships. No symptom indicative of temporomandibular joint disease was detected in either of the patients.

Despite their similarities, the twin patients also exhibited distinct facial and intraoral differences, including features that demonstrated mirror symmetry. Patient 1 presented an approximately 3-mm leftward deviation of the chin, with the right side of the face appearing fuller compared to the left. Both the upper and lower dental midlines shifted approximately 2 mm to the left relative to the facial midline. The maxillary lateral incisors were positioned palatally, resulting in a crossbite relationship, while the maxillary canines were positioned labially. The lower dentition displayed no crowding, with slight spacing observed in the region of the second premolars. All four first molars exhibited fillings on their occlusal surfaces. Patient 2 presented with a rightward chin deviation of approximately 3 mm, with the left side of the face appearing more prominent. The upper dental midline deviated approximately 3 mm to the right, while the lower deviated around 2 mm to the right relative to the facial midline. The maxillary right lateral incisor was positioned palatally and exhibited a crossbite relationship, while the canines were located labially. Mild crowding was observed in the lower dentition.

**Figure 2:**
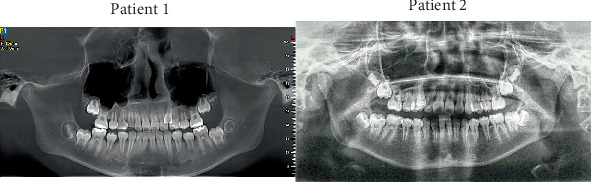
Pretreatment panoramic radiographs.

**Figure 3:**
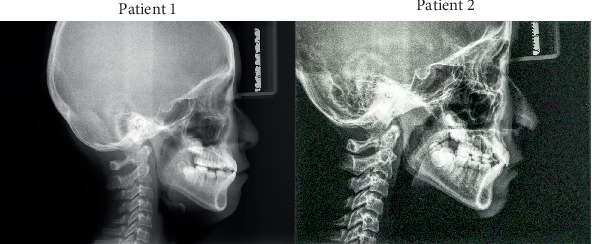
Pretreatment lateral cephalograms.

Pretreatment panoramic radiographs (Figure 2) confirmed that both patients had a full complement of teeth, including four developing third molars. In Patient 2, the occlusal surfaces of all four first molars exhibited radiopacity. Pretreatment lateral cephalometric analyses (Figure 3 and Table 1) indicated a skeletal Class I relationship with a normal mandibular plane angle in both patients.

Both patients were diagnosed with Class II malocclusion, characterized by a balanced facial profile, an average mandibular plane angle, and dental crowding.

The treatment objectives for both patients were essentially the same, focusing on alleviating crowding, aligning the dentition, and achieving proper occlusion, thereby improving both smile esthetics and occlusal function.

Prior to formulating the final treatment plans, a comprehensive analysis of the data from both cases was conducted, and various potential approaches were systematically evaluated. Severe crowding in the upper dentitions of both patients necessitated maxillary extractions. In Patient 1, both maxillary and mandibular incisor inclinations fell within the normal range, accompanied by a moderate amount of space in the lower dentition. These clinical features supported the feasibility of utilizing the two-premolar extraction pattern to achieve the treatment objectives. The key distinctions were that Patient 2 presented with certain labial inclination of the mandibular incisors and mild crowding in the lower dentition. Under these conditions, achieving the desired treatment outcomes would not have been possible without mandibular extractions. These considerations led to the finalization of distinct orthodontic treatment protocols for the identical twins. For Patient 1, the plan involved extracting two maxillary first premolars, while for Patient 2, the extraction of all four first premolars was required. In consultation with the patients' guardians, it was explicitly conveyed that the two-premolar extraction pattern could risk treatment stagnation. Should the progress be impeded, the removal of an additional two mandibular premolars would be required to ensure the advancement of the treatment.

Before the commencement of orthodontic tooth movement, all required extractions were completed for both patients, followed by the bonding of McLaughlin–Bennett–Trevisi prescription brackets. Alignment and leveling took approximately 7 months for each patient. Both followed a standard archwire sequence, ending with 0.018 × 0.025-in. stainless steel wires for space closure. The key differences lay in anchorage preparation and space closure strategies. In Patient 1, maximum anchorage was prepared in the mandible to allow for retraction of the anterior teeth. Pre-existing spacing and mild arch expansion during alignment provided sufficient space for retraction, resulting in a normal anterior overjet. In the maxilla, space closure was achieved with minimum anchorage, and mesial movement of the posterior teeth was aided by Class III elastics. In Patient 2, the residual spaces in both the upper and lower dentitions were closed using a reciprocal anchorage mechanism. The treatment duration for Patient 1 spanned approximately 29 months, while that for Patient 2 lasted around 35 months. Upon completion, all brackets were debonded, and vacuum-formed retainers were provided for retention. Full-time wear was recommended for the first year, followed by at least 1 year of night-time wear.

**Figure 4:**
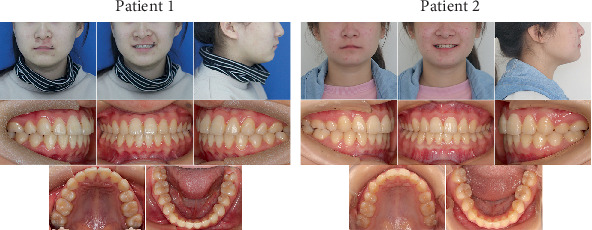
Posttreatment facial and intraoral photographs.

Posttreatment facial and intraoral photographs (Figure 4) taken for the two patients provided compelling evidence that the treatment objectives were fully attained. The commonalities included well-aligned dentitions, harmonious intercuspal occlusion, and enhanced smile esthetics, along with centered midlines, bilateral Class I canine relationships, and optimal anterior overbite and overjet. In Patient 1, the extraction of two maxillary first premolars led to the establishment of a bilateral complete Class II molar relationship upon treatment completion. In contrast, following the symmetrical extraction of four first premolars, Patient 2 attained a bilateral Class I molar relationship.

**Figure 5:**
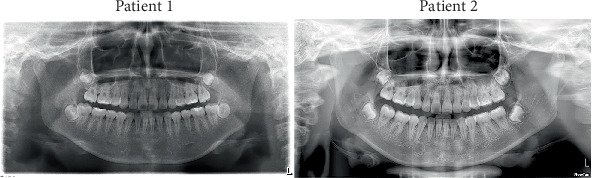
Posttreatment panoramic radiographs.

**Figure 6:**
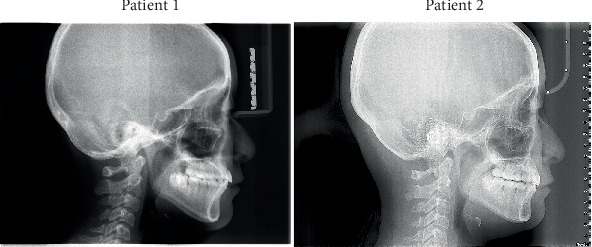
Posttreatment lateral cephalograms.

Posttreatment panoramic radiographs (Figure 5) obtained for both patients confirmed favorable treatment outcomes, with well-aligned teeth, appropriate root parallelism, and complete space closure, all without any indication of marginal bone loss or root resorption. Posttreatment lateral cephalometric analyses (Figure 6 and Table 1), in comparison with pretreatment values, revealed that Patient 1's anterior overbite and overjet increased to within the normal range. This improvement was primarily attributed to the retraction of mandibular anterior teeth (IMPA decreased from 98.3° to 93.8°). As a recipient of the four-premolar extraction pattern, Patient 2 exhibited a measurable retraction of both maxillary and mandibular anterior teeth following treatment (U1-SN decreased from 102.2° to 96.6°, IMPA decreased from 102.9° to 96.9°). Importantly, both the overjet and overbite remained within clinically acceptable parameters. In Patient 1, a mild clockwise mandibular rotation was noted, with the SN-MP angle increasing from 39.1° to 41.5°, likely due to the uncontrolled eruption and distal tipping of the second molars during treatment.

**Figure 7:**
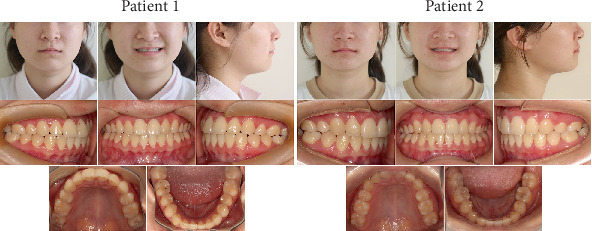
Facial and intraoral photographs taken at the 4-year follow-up.

Follow-up records from both patients (Figure 7), taken about 4 years after completing orthodontic treatment, confirmed the long-term stability of the outcomes, including proper tooth alignment, functional occlusion, and a more refined smile.

## 3. Discussion

Considering the substantial influence of genetics on the etiology of the condition, the treatment outcomes in identical twins serve as an invaluable reference for concurrent controlled trials, facilitating a more accurate assessment of treatment effects by minimizing genetic variability [17]. The debate regarding long-term stability of two- versus four-premolar extraction protocols has persisted for many years. The escalation of this debate can be attributed to concerns that completing orthodontic treatment with a Class II molar relationship may result in increased posttreatment instability [18]. A comparative study featuring identical twins could yield robust evidence to guide clinical consensus and influence the interpretation of long-term treatment outcomes in this context. Over a comprehensive 4-year follow-up period following treatment, no significant alterations were observed in terms of tooth alignment, occlusal relationship, or facial profile in this set of identical twins. The long-term monitoring of this pair of cases supports the conclusion that both two- and four-premolar extraction patterns can achieve sustained stability, provided that appropriate clinical indications are carefully selected [19].

The four-premolar extraction protocol represents a well-established and highly refined orthodontic technique. Its clinical indications are well understood and have been expertly integrated into practice by the vast majority of orthodontic professionals. Although the two-premolar extraction protocol is commonly employed, there remains a lack of consensus within the orthodontic community regarding the specific types of malocclusion for which it is considered the most effective treatment option. The authors of this article contend that the two-premolar extraction protocol may be a preferable option over the four-premolar extraction protocol under the following conditions: (1) Both the maxilla and mandible exhibit normal positioning and size, or there is no significant maxillomandibular discrepancy. (2) The molar relationship is either a complete or nearly complete Class II. (3) The upper dentition exhibits severe crowding, or the maxillary anterior teeth are significantly proclined. (4) The lower dentition shows no notable crowding, excessive deepening of the curve of Spee, or significant proclination of the anterior teeth. (5) The upper and lower arch widths match. The malocclusion in Patient 1 basically met all the aforementioned criteria, allowing for the successful completion of orthodontic treatment with extraction of only two maxillary first premolars and achieving favorable long-term stability. The mandibular anterior teeth in Patient 2 exhibited certain labial inclination, accompanied by a degree of crowding in the lower dentition, rendering two-premolar extraction protocol unsuitable for this case.

The other key distinctions between two- and four-premolar extraction protocols in managing Class II malocclusion lie in anchorage design and treatment duration required. Studies have shown that achieving correction of Class II malocclusion through the extraction of two maxillary premolars yields a higher rate of occlusal success compared to the conventional four-premolar extraction method. This outcome is attributed to the greater anchorage reinforcement required to establish Class I molar and canine relationships, which often involves removable appliances and relies heavily on patient compliance [20]. Additionally, two-premolar extraction protocol typically results in shorter treatment times than four-premolar extraction approach, as the correction of molar and canine relationships in the latter inherently lengthens the durations [13, 14]. For Patient 2, considerable time was dedicated to closing the extensive spaces in the lower dentition and maintaining anchorage control throughout the process. Furthermore, prolonged use of Class II elastics was required to correct the molar relationship, which presented a significant challenge in terms of patient compliance. In contrast, after resolving the crowding in the upper dentition, Patient 1 had scarcely any space remaining, and intermaxillary elastics were almost unnecessary throughout the treatment. This resulted in a shortened treatment duration, with a reduced dependency on patient compliance, similarly leading to favorable treatment outcomes.

Moreover, it is worth noting that in cases where third molars are present, four-premolar extraction treatment is associated with a reduced risk of mandibular third molar impaction compared to the two-premolar extraction protocol. Prior to treatment, the mandibular third molars of the identical twins exhibited nearly identical positions and angulations. However, upon reviewing the posttreatment panoramic radiographs, it is evident that Patient 1's mandibular third molars have developed a distinct mesial impaction, whereas Patient 2's appear to have adequate eruption space. This difference can be attributed to the mesial movement of posterior teeth during mandibular premolar space closure, which alleviated the impaction in Patient 2. Following treatment, we recommended the early removal of Patient 1's third molars, as impaction could pose a potential risk for future crowding.

One final caveat, this article only presents a comparison and analysis of a single set of identical twins, limiting the findings to individual cases. As such, the conclusions may not be generalizable in comparison to large-scale, controlled clinical trials. Additional large-scale clinical trials are needed to evaluate two- and four-premolar extraction protocols, providing more objective data and enhancing the external validity and reliability of the conclusions.

## 4. Conclusion

This case report concludes that both two- and four-premolar extraction protocols can achieve ideal treatment results in terms of tooth alignment, occlusion, and facial profile. However, their indications differ, with the two-premolar extraction protocol being suitable for more specific cases. When appropriately selected, the two-premolar extraction protocol can achieve comparable long-term stability, with reduced invasiveness, a shorter treatment duration, and a higher occlusal success rate.

## Figures and Tables

**Table 1:** Cephalometric measurements.

**Measurement**	**Normal**	**Patient 1**		**Patient 2**	
		**Pretreatment**	**Posttreatment**	**Pretreatment**	**Posttreatment**
Skeletal					
SNA (°)	82.8 ± 4.1	75.1	73.8	73.7	73.9
SNB (°)	80.1 ± 3.9	73.2	71.2	70.8	72.0
ANB (°)	2.7 ± 2.0	1.9	2.6	2.9	1.9
FMA (°)	27.3 ± 6.1	29.8	30.1	28.1	27.0
SN-MP (°)	30.4 ± 5.6	39.1	41.5	39.8	38.0
Dental					
U1-SN (°)	105.7 ± 6.3	104.9	104.3	102.2	96.6
IMPA (°)	96.7 ± 6.4	98.3	93.8	102.9	96.9
Overjet (mm)	2.0 ± 1.0	0.9	3.0	2.3	2.3
Overbite (mm)	3.0 ± 2.0	0.2	1.6	1.0	1.0
Soft tissue					
UL-EP (mm)	−1.4 ± 0.9	−0.5	−1.2	0.6	−1.7
LL-EP (mm)	0.6 ± 0.9	1.2	−1.0	2.4	−1.6

Abbreviations: ANB, A point–nasion–B point; FMA, gonion–menton to Frankfort horizontal plane; IMPA, lower incisor to gonion–menton; LL-EP, lower lip to E line; SNA, sella–nasion–A point; SNB, sella–nasion–B point; SN-MP, sella–nasion to mandibular plane; U1-SN, upper incisor to sella–nasion; UL-EP, upper lip to E line.
